# Typical viewpoints of objects are better detected than atypical ones

**DOI:** 10.1167/jov.22.12.1

**Published:** 2022-11-01

**Authors:** Evan G. Center, Austin M. Gephart, Pei-Ling Yang, Diane M. Beck

**Affiliations:** 1 Beckman Institute, University of Illinois Urbana-Champaign, Urbana, IL, USA; 2 Psychology Department, University of Illinois Urbana-Champaign, Urbana, IL, USA

**Keywords:** object perception, statistical regularity, predictive coding

## Abstract

Previous work has claimed that canonical viewpoints of objects are more readily perceived than noncanonical viewpoints. However, all of these studies required participants to identify the object, a late perceptual process at best and arguably a cognitive process (Pylyshyn, 1999). Here, we extend this work to early vision by removing the explicit need to identify the objects. In particular, we asked participants to make an intact/scrambled discrimination of briefly presented objects that were viewed from either typical or atypical viewpoints. Notably, participants did not have to identify the object; only discriminate it from noise (scrambled). Participants were more sensitive in discriminating objects presented in typically encountered orientations than when objects were presented in atypical depth rotations (Experiment 1). However, the same effect for objects presented in atypical picture plane rotations (as opposed to typical ones) did not reach statistical significance (Experiments 2 and 3), suggesting that particular informative views may play a critical role in this effect. We interpret this enhanced perceptibility, for both these items and good exemplars and probable scenes, as deriving from their high real-world statistical regularity.

## Introduction

Over the past 3 decades, mounting evidence calls for updating, or even replacing, the serial model of visual perception (Rubin, 1915; Wertheimer, 1923/1938; Palmer & Rock, 1994; Nakayama, He, & Shimojo, 1995; Driver & Baylis, 1996) with a recursive one. Peterson and colleagues demonstrated that participants are more likely to identify meaningful regions of images as figure rather than ground (Peterson, Harvey, & Weidenbacher, 1991; Gibson & Peterson, 1994; Peterson & Gibson, 1994), even when researchers presented images at extremely brief durations followed by masks (28 ms; Gibson & Peterson, 1994). These results were among the first to suggest that prior knowledge plays a role in basic perception. Later, Grill-Spector and Kanwisher (2005) showed that categorizing a natural image occurs in the same time frame as simply detecting the presence of a natural image, suggesting that observers can categorize images as soon as they can detect that a coherent image is present. Although we note that this was only true for comparisons in which stimuli derived from the same superordinate-level class (Mack & Palmeri, 2010), suggesting that rather than categorization and detection co-occurring, some categorizations and detections might rely on information that becomes available at the same time.

We, however, have evidence that category information (not categorization per se) impacts detection. Participants were better at detecting the presence of good exemplars of a natural scene category than bad exemplars of their category (Caddigan, Choo, Fei-Fei, & Beck, 2017). Researchers presented either intact or phase-scrambled images of natural scenes, followed by a mask, and asked participants to report whether the image preceding the mask was intact or scrambled. Participants were more accurate at discriminating good scene exemplars from noise than bad scene exemplars. It is critical to note that the referenced task did not require participants to identify or categorize the stimuli; only to report whether they were seeing an intact natural scene or noise, thus making a case that the effect is perceptual as opposed to conceptual. Although participants are not required to categorize or identify the image, it seems its identity or the degree to which it exemplifies its category nonetheless influences how readily it is perceived. These data create difficulty for models that assume that recognition and categorization can occur only after detection.

Perhaps then, counterintuitively, something must be recognized before one can even know that it is there. Canonicity has been shown to impact recognizability, and we in turn predict that canonicity, or in practice, presenting an object from its canonical viewpoint, should also result in better detection than a noncanonical viewpoint. Palmer, Rosch, and Chase (1981) defined canonical viewpoints by asking participants to rate the goodness of object viewpoints, to imagine objects, or to photograph objects, and found that canonical viewpoints defined in any aforementioned manner were correlated with faster naming of those objects. The researchers interpreted their findings as evidence that the brain stores long term memories for objects from their most informative viewpoints, and that the extent to which new exemplars make contact with this canonical viewpoint determines a participant's ability to efficiently identify it. Blanz, Tarr, and Bülthoff (1999) argue that what makes an object viewpoint canonical comes down to a combination of factors, including familiarity, functionality, and geometry, with familiarity doing most of the “heavy lifting.”

**Figure 1.**
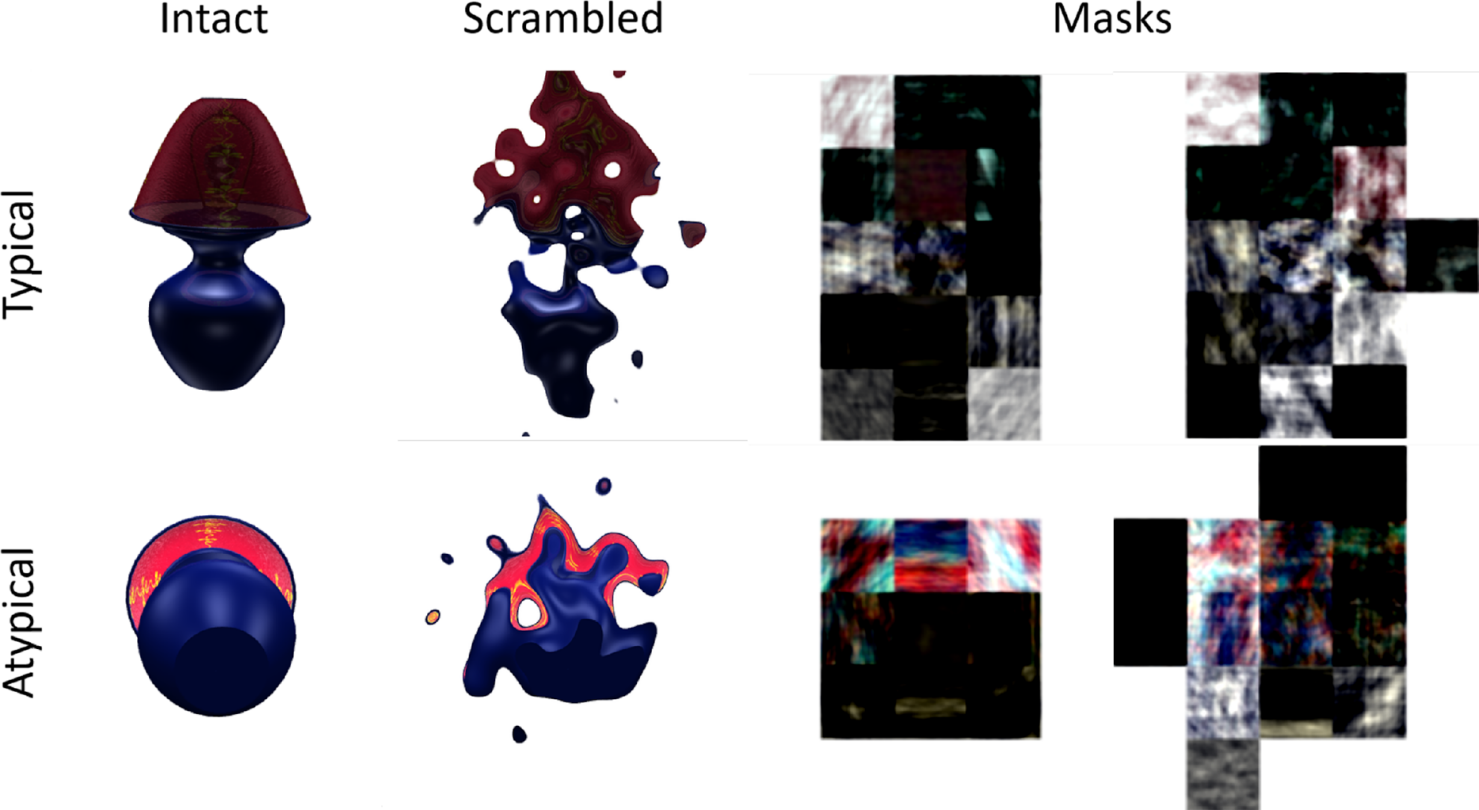
Stimuli presented in Experiment 1. Top row shows typical object viewpoints. Bottom row shows atypical object viewpoints. First column shows intact objects. Second column shows scrambled (diffeomorphed) objects. Third column shows box-scrambled masks for intact objects. Fourth column shows box-scrambled masks for scrambled (diffeomorphed) objects.

Although research investigating canonicity often talks about canonical viewpoints affecting perception, to date, that research relies overwhelmingly on object identification (e.g. Lawson & Humphreys, 1996; Hayward & Tarr, 1997; Lawson & Humphreys, 1998). Pylyshyn (1999) has argued, however, that identification is a cognitive rather than a perceptual process. Thus, under this logic, the effects of canonicity thus far can be said to affect the participants ability to label the object, rather than their ability to perceive it. Here, we make an even more fundamental claim: that canonical viewpoints are more readily perceived in an early vision sense. Rather than ask participants to name objects or discriminate studied from unstudied objects, here, they are simply asked whether what they were shown was an object at all. On each trial, participants indicated whether the item flashed on the screen was intact or scrambled. Trials displayed either an intact object, viewed from a typical or atypical orientation, or a meaningless diffeomorph (a splatted, non-object transformation of an object from the same set of intact objects; Stojanoski & Cusack, 2014). Although the scrambled objects are centralized in the frame like the real objects, they differ considerably from intact objects (see Figure 1). Thus, in principle, one could determine whether something was intact or scrambled without having to recognize the intact object. Certainly, with extended viewing it is trivial to determine which images are objects and which are not, even when one might be unclear about the objects’ identity. Importantly, we titrated presentation duration to each participant's 82% accuracy threshold. Under such brief presentations, participants’ phenomenology is typically, but not always, of seeing just a flash and guessing. We then examined detection sensitivity for intact object trials as a function of whether the object was shown in a typical or atypical orientation. If, under very brief presentations, participants are better able to discriminate canonical viewpoints than noncanonical viewpoints, we take this to mean that they more readily “perceive” canonical viewpoints.

One notable exception to the theme of using explicit identification tasks to test the role of viewpoint canonicity in object perception comes via the work of Srinivas (1995). In parts of this experiment, non-objects were constructed by “pasting different parts of familiar objects together,” and participants were presented with a stimulus in canonical or unusual depth rotations for 3 seconds on each trial. Participants were tasked with indicating whether the stimulus was an object or non-object, and the effect of viewpoint orientation was measured in terms of response latency costs. Whereas numerically greater response latencies for unusual relative to canonical views for unstudied objects were reported, these results were not further analyzed as the primary theoretical interest of this work lied in priming effects stemming from previously studied objects. Furthermore, due to the relatively long exposure durations used, one could still argue that a cognitive rather than perceptual process is responsible for the effect, despite the simple discrimination task used. Thus, as in the analogous experiment by Caddigan et al. (2017) in which good exemplars of scenes were better detected than bad exemplars, in the present experiment, we are predicting that detection is affected by factors that impact recognizability; that is, representativeness in the case of Caddigan et al. (2017) and canonicity of viewpoint in the experiments presented here. Although, as argued above, in neither case is the task to explicitly identify the stimulus.

In Experiment 1, we predicted that typically oriented object viewpoints would be discriminated from noise more readily than atypically oriented objects. Experiment 1 was preregistered on Open Science Framework (https://osf.io/ytp5d). Experiment 1 involved rotations in depth which changed many of the features present among canonical and noncanonical orientations, described in Palmer, Rosch, and Chase (1981) as those informative, salient features that support efficient object identification. We explore this issue in Experiment 2 by rotating objects in the picture plane, thus preserving all visible information between canonical and noncanonical viewpoints other than orientation. If the frequency with which we encounter objects in these orientations is what drives our ability to detect them, we should still observe a drop in sensitivity to these objects, but if the presence or absence of key features affecting recognizability is more critical in object detection, we may not observe a drop in sensitivity. Experiment 3 then replicates the findings of Experiment 2 using a larger sample based on a power analysis of Experiment 2.

## Experiment 1

### Methods

Experiment 1 sought to conceptually replicate previous findings regarding better perception for more easily recognized items. We probed whether this effect would generalize beyond natural scenes and predict the pattern of discrimination for isolated objects, whereby typically oriented objects (seen from canonical viewpoints) would be better discriminated from noise than atypically oriented objects (those same objects now rotated in depth to make their viewpoint noncanonical).

### Participants

Participants were recruited from the University of Illinois participant pool and compensated in partial course credit. All had normal or corrected-to-normal vision. Participants were given written informed consent in accordance with procedures and protocols approved by the University of Illinois Institutional Review Board. We collected data from 23 participants in a pilot experiment in order to estimate an effect size and determine the size of our experimental sample. Our pilot experiment resulted in an effect size reflecting greater sensitivity for typically oriented than atypically oriented stimuli corresponding to a Cohen's *dz* of 0.78. We used G*Power software (http://www.psychologie.hhu.de/arbeitsgruppen/allgemeine-psychologie-und-arbeitspsychologie/gpower.html) to perform a power analysis and selected a sample size of 20 participants in order to target 95% power to detect an effect of equal or greater magnitude to our pilot's effect size.

### Stimuli and procedure

Stimuli were images of 180 unique household objects isolated over white backgrounds, in two orientations each, one typical and one atypical, for a total of 360 images. Each object was shown once in each orientation in the experiment. Images were acquired via the Tarr Lab Object Databank (http://wiki.cnbc.cmu.edu/images/TheObjectDatabank.zip). The dimensions of each image were 450 by 450 pixels and subtended roughly 11.93 degrees by 11.93 degrees of visual angle. Viewpoints were selected by experimenters among a variety of available viewpoints and later verified as typical or atypical by participant ratings. Typical viewpoints were selected to depict objects in upright, frequently encountered orientations, whereas atypical viewpoints were selected to depict objects in one of various infrequently encountered depth rotations, among those available within the Tarr Lab Object Databank. The typical and atypical designations were confirmed by participant ratings (see Results).

Because images contained isolated objects over white backgrounds, we opted to use 50% “diffeomorphed” objects (Stojanoski & Cusack, 2014; see report for details on their novel method) as our scrambled images (see Figure 1) rather than phase scrambling the images. The diffeomorphing technique is similar to phase scrambling in that low-level information remains intact while high-level information, such as item identity, is distorted, but unlike phase scrambling, the algorithm only operates on the object itself rather than the full image, allowing us to create “scrambled” objects that are less easily discriminated from intact images. One might describe a diffeomorphed object as a somewhat “melted” version of the intact original. Layers of the HMAX computational model of object recognition designed to model early visual processing have shown indistinguishable responses to intact and diffeomorphed images, whereas later layers in the model successfully distinguish between the two image classes (Stojanoski & Cusack, 2014).

Phase scrambling would have distributed the contents of the objects evenly across the whole image, rendering them too easy to discriminate from isolated objects on a uniform background, although we did employ a form of phase scrambling in creating masks. Similarly, general 1/F noise masks covering the entirety of stimulus dimensions were found to be ineffective at masking isolated objects in early tests. We instead created unique item masks by “box scrambling” (Vogels, 1999) each intact and diffeomorphed object which we have found to provide effective masking. To do so, we applied an invisible five by five grid over each image and performed traditional phase scrambling independently for each portion of the grid, thus creating noisy masks of comparable spatial extent to each object. Stimuli and instructions were presented on an 85 Hz monitor of resolution 1280 by 960 using PsychoPy (Pierce, 2007; Pierce, 2009) and Python software (Python Software Foundation. Python Language Reference, version 2.7 [Experiment 1] and version 3.7 [Experiments 2 and 3]. Available at http://www.python.org).

Participants were seated comfortably in a chinrest 59 cm from the monitor. In addition to written instruction, participants were also given verbal instruction that, on each trial, an object would briefly appear on the screen followed by a mask and that their task would be to determine whether the object was intact or scrambled, pressing one control key (on a standard keyboard) if they think the object is intact, and the other control key if they think the object is scrambled (counterbalanced among participants). Participants were asked to respond as quickly and accurately as possible, making their best guess if they could not tell whether the object was intact or scrambled. Each participant was shown an example of an intact and a scrambled object before beginning trials (an intact rubber duck and diffeomorphed version of the rubber duck, which was the same for all participants). Because performance varies considerably on this task, durations were determined separately for each participant. Thus, each participant began with 240 staircasing trials, presented over eight blocks, in which we used the Quest algorithm (Watson & Pelli, 1983) to derive a stimulus presentation duration, separately for each participant, which produced 82% performance accuracy, roughly equivalent to a three up one down staircase. Stimuli for staircasing (120 intact and 120 scrambled) were selected randomly from the full set (360 intact and 360 scrambled) but were not re-used in the main experiment. Stimulus types were randomly intermixed on each trial throughout the staircasing procedure, forming a single staircase.

**Figure 2.**
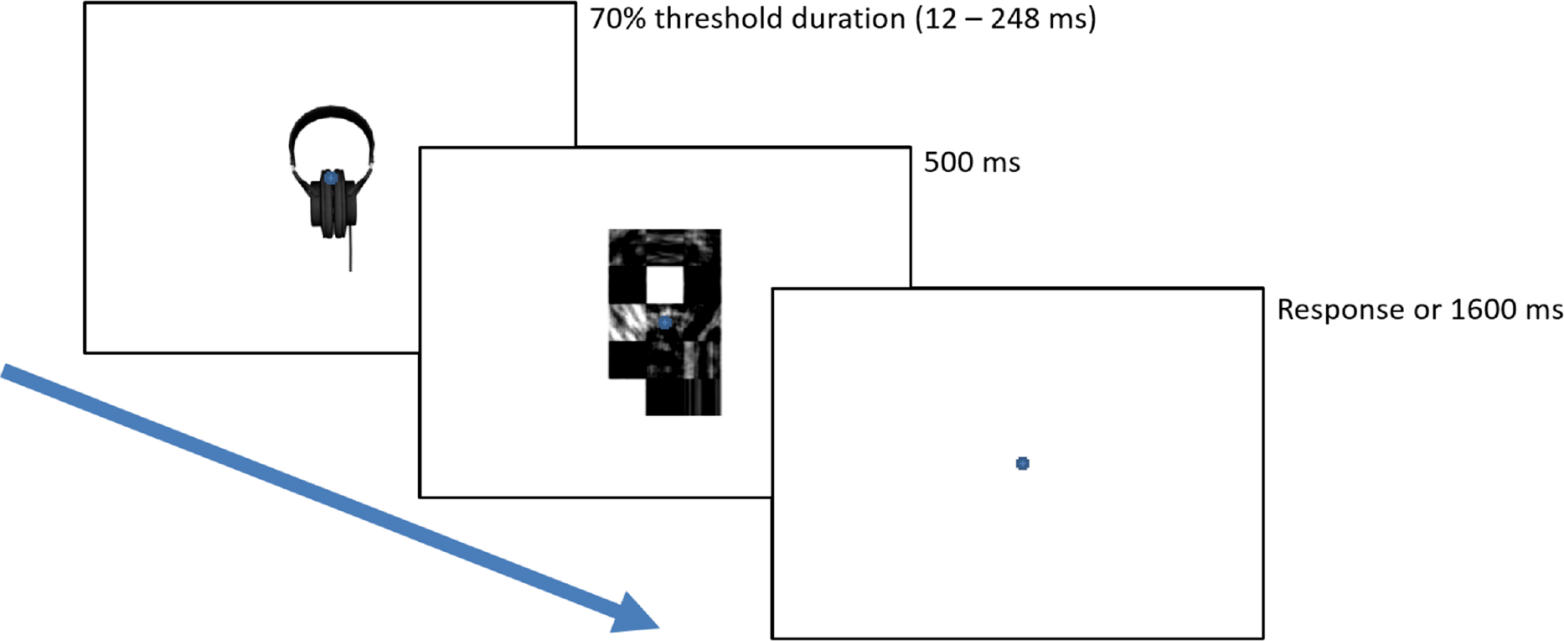
Procedure for Experiments 1 and 2. Participants attended a fixation then were presented with either an intact or scrambled object in either a typical or atypical orientation for a duration between 12 and 248 ms, staircased to 82% accuracy for each individual. Each object was then followed by its own box-scrambled mask for 500 ms. Participants then had 1600 ms to make a response before a 500 to 1500 ms inter-trial interval began.

Participants then underwent 480 main trials presented over 16 blocks. Participants were encouraged to take short breaks between blocks. The order of all object presentations was randomized. Each trial in the staircasing and main tasks consisted of two sequentially presented images: first either an intact or scrambled object, then that object's box scrambled mask. If the participant failed to respond within 1.5 seconds, the response was counted as incorrect, and the next trial began (see Figure 2). The duration of the object (minimum possible duration = 12 ms; and maximum needed = 247 ms) in each trial was determined by the Quest algorithm for each participant, and each mask lasted 500 ms, all frame-locked to an 85 Hz display. There was a 500 to 1500 ms random delay between the end of one trial (whether by keypress or failure to respond) and the beginning of the next. After the main blocks, participants also rated a random subset of 240 stimuli as to how typical each viewpoint of each object appeared to them using a 7-point Likert-like scale, with 1 corresponding to highly atypical and 7 corresponding to highly typical.

### Data analysis

Participants who failed a chi-square test at significance level α = 0.05 for accuracy above chance level and those who scored below 50% accuracy in discrimination performance were excluded from further analysis. Trials containing response times less than 50 ms after the termination of the mask were considered premature and discarded. We performed a one-tailed within-participant *t*-test for *d*-prime on the remaining participants to test the hypothesis that typically oriented objects are better discriminated than atypically oriented objects, per our preregistered analysis plan. Any follow-up analyses not included in the pre-registration were performed as two-tailed tests.

### Results and discussion

**Figure 3.**
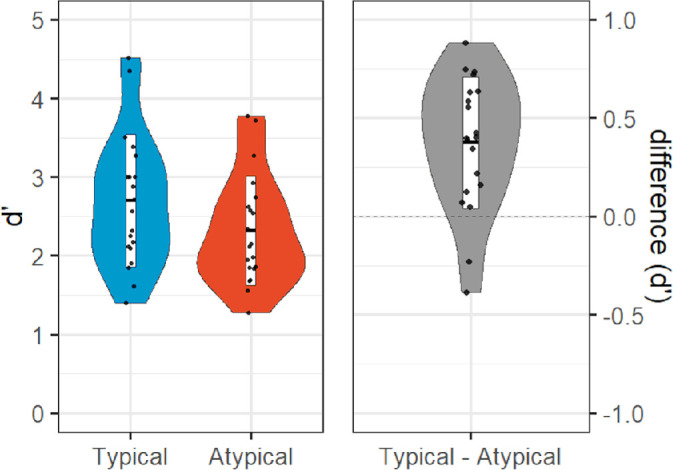
Experiment 1 (depth rotation) results: violin plots of sensitivity (*d*-prime) to typical (left within left panel) and atypical (right within left panel) objects, and difference (right panel).

**Figure 4.**
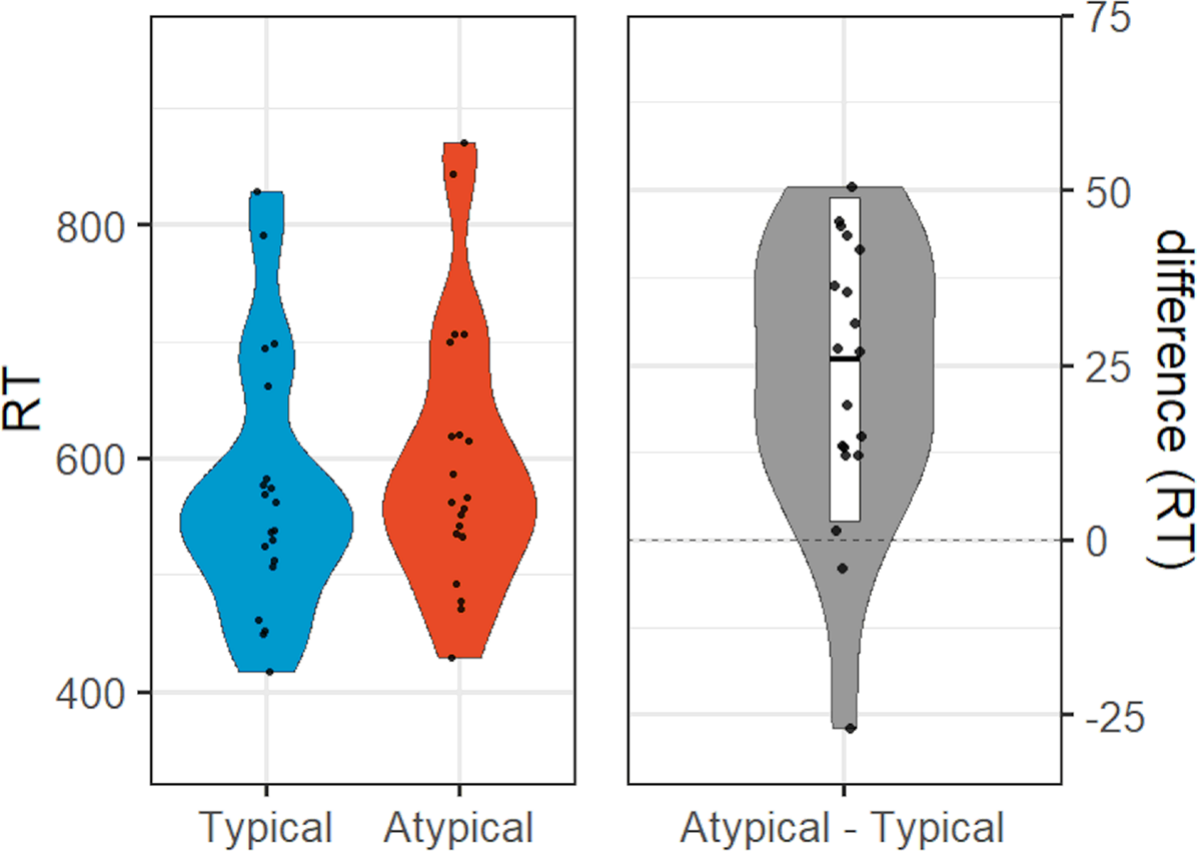
Experiment 1 (depth rotation) results: violin plots of reaction times in milliseconds for typical (left within left panel) and atypical (right within left panel) objects, and difference (right panel).

No participants met the exclusion criteria for Experiment 1. On average, participants required eight frames (approximately 94 ms) to attain an 82% accuracy threshold as determined by the Quest algorithm (*SD* = 6 frames or approximately 71 ms). A one-tailed within-participant *t*-test on *d*-prime indicated that typically oriented objects (*M* = 2.70, *SD* = 0.84) were better discriminated than atypically oriented objects (*M* = 2.32, *SD* = 0.70), *t*(19) = 4.99, *p *< 0.001, *dz* = 1.12; Figure 3). Although preregistered hypotheses were only made with respect to *d*-prime, participant performance was also more accurate for typically oriented objects (*M* = 0.86, *SD* = 0.09) than atypically oriented objects (*M* = 0.79, *SD* = 0.13), *t*(19) = 4.88 (two-tailed), *p *< 0.001, *dz* = 1.09, and response times were faster for typically oriented objects (*M* = 573 ms, *SD* = 111 ms) than atypically oriented objects (*M* = 599 ms, *SD* = 116 ms), *t*(19) = −5.01 (two-tailed), *p *< 0.001, *dz* = −1.12; Figure 4). Confirming our initial classification of our objects, our participants rated typically oriented objects (*M* = 5.46, *SD* = 1.52) as more highly typical viewpoints than atypically oriented objects (*M* = 3.25, *SD* = 0.93), significant in both parametric (*t*(19) = 6.38 (two-tailed), *p *< 0.001, *dz* = 1.43) and nonparametric (Wilcoxon signed rank test; *Z* = 3.62, *p* < 0.001) tests.

Rating results from Experiment 1 thus confirm that participants saw objects we designated as typical as more highly typical viewpoints than objects we designated as atypical. Furthermore, our intact versus scrambled judgment results suggest that object viewpoint typicality influences early perceptual processes, in agreement with previous manipulations of recognizability for other stimuli.

## Experiment 2

Experiment 2 tests the degree to which the effect observed in Experiment 1 depends on rotation in depth and thus may be better described as an effect of canonical viewpoint; that is, a viewpoint in which the most information regarding object identity is visible. We dissociated typicality of viewpoint from canonicity by taking our typically oriented (in depth) objects and rotating them in the picture plane instead. If the detection effect is purely determined by how often we encounter these viewpoints, we should expect the same result as Experiment 1 because we do not commonly encounter these objects in our chosen atypical orientations. However, if particular views provide more information with respect to what the object is, in keeping with a canonical viewpoint, then a rotation in the picture plane leaves that information intact and so we would expect a weak effect of that rotation on detection. The work of Jolicoeur and colleagues (e.g. Jolicoeur, 1985; Lawson & Jolicoeur, 1998; Lawson & Jolicoeur, 1999; Lawson & Jolicoeur, 2003) captures well the link between object orientation and subsequent *identification* latency and accuracy for familiar objects, demonstrating that response latencies and identification errors rise as objects are rotated further from their upright position. Similar results regarding the relationship between object orientation and identification have been described by Tarr and Pinker (1989) and Tarr (1995) even for novel objects which participants were given ample time to study prior to testing. Experiment 2 asks whether these types of effects extend beyond identification. In a detection task identical to that of Experiment 1, we present familiar, unstudied objects in atypical (picture-plane rotated) orientations to examine their impact on basic perceptual processes.

### Methods

Methods for Experiment 2 were identical to those of Experiment 1 except that images used in Experiment 2 were rotated in the picture plane rather than in depth; that is, the canonical objects were presented either upright or rotated by 180 degrees, or less frequently, 90 degrees. Rotation angles were selected by experimenters per object and later confirmed by participant ratings as atypical. Typical viewpoints were selected to depict objects in upright, frequently encountered orientations, whereas atypical viewpoints were selected to depict objects non-upright, infrequently encountered orientations. The vast majority of atypical viewpoints were 180 degree rotations of their upright counterparts, however, a minority of atypical viewpoints received 90 degree rotations instead due to the nature of the stimulus (e.g. one of these minority instances was a tall filing cabinet where a 90 degree rotation created an infrequently encountered viewpoint, but a 180 degree rotation could only be discriminated from its upright counterpart by paying careful attention to the orientation of the handles on the file drawers). Again, typicality designations were confirmed by participant ratings (see Results). Data from 20 participants were collected. A subset consisting of 240 of the original 360 images were used (comprising of 120 upright objects and 120 rotations of those same objects), which excluded most oblong objects that do not have atypical rotations in the picture plane (consider a pencil, which has no atypical rotation in the picture plane, compared to a tall filing cabinet, which does) and objects with radial symmetry, as none of the picture plane rotations for these objects look particularly atypical. Thus, participants performed 180 Quest trials followed by 300 main trials, then 240 rating trials for viewpoint typicality.

### Results and discussion

**Figure 5.**
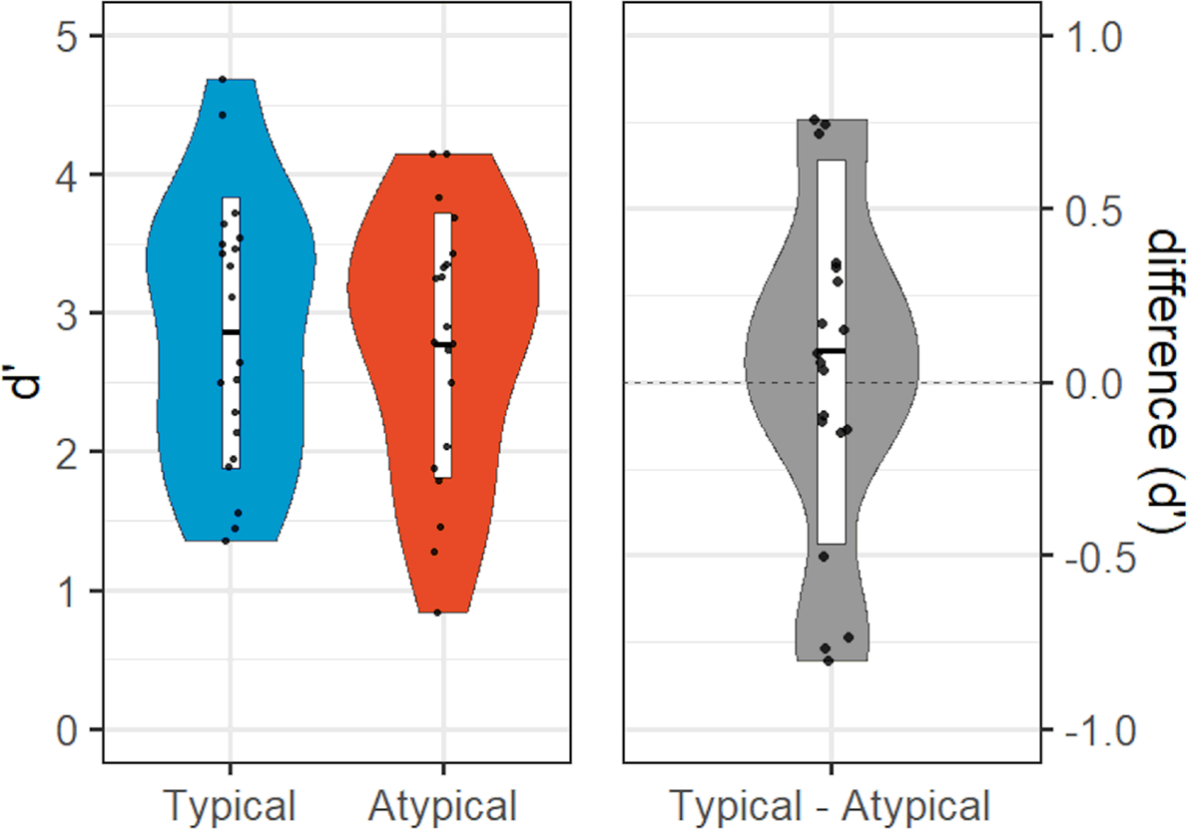
Experiment 2 (picture-plane rotation) results: violin plots of sensitivity (*d*-prime) for typical (left within left panel) and atypical (right within left panel) objects, and difference (right panel).

**Figure 6.**
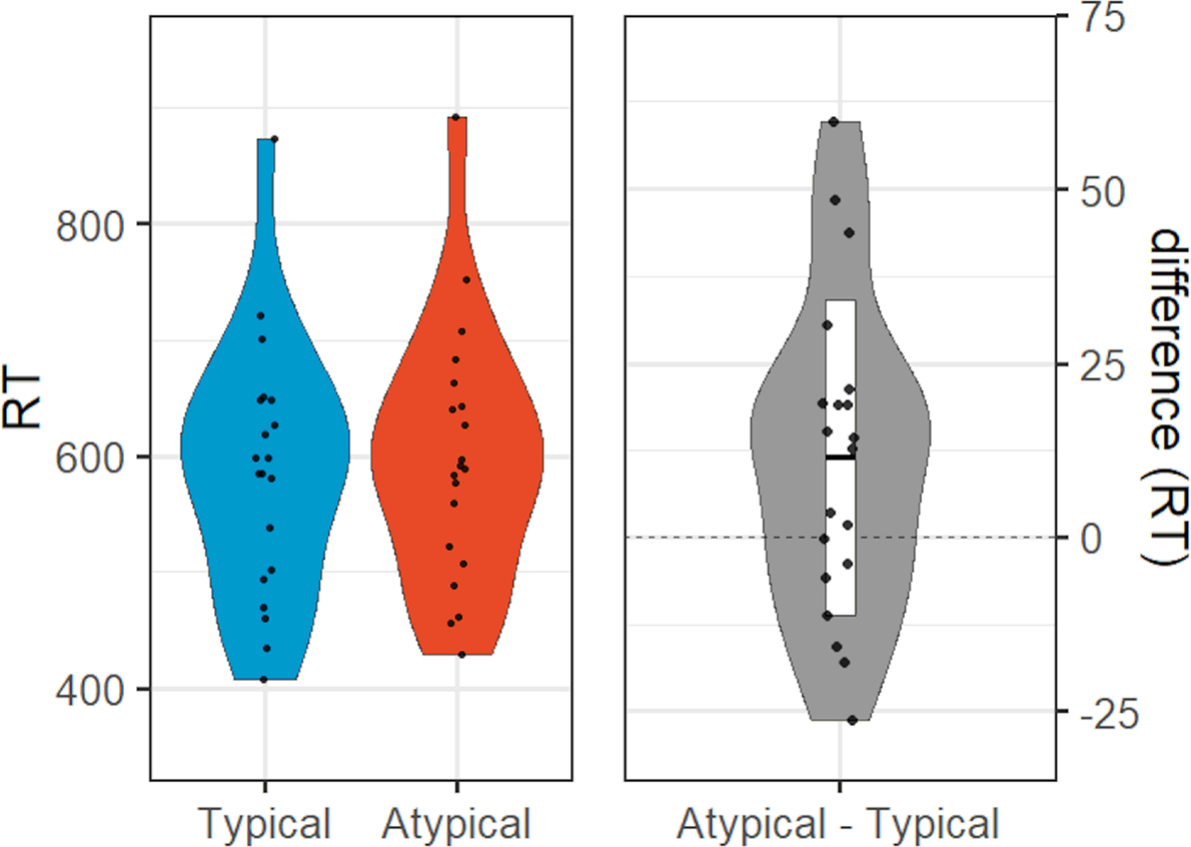
Experiment 2 (picture-plane rotation) results: **v**iolin plots of reaction times in milliseconds for typical (left within left panel) and atypical (right within left panel) objects, and difference (right panel).

We excluded six participants from analyses for Experiment 2, due to either computer malfunction (*n* = 2) or the participant responding randomly and failing our chi-square criterion (*n* = 4). New data were collected to replace these participants before further analyses were performed. On average, participants required 10 frames (approximately 118 ms) to attain an 82% accuracy threshold as determined by the Quest algorithm (*SD* = 5 frames or approximately 59 ms). Atypically oriented objects created via rotations in the picture plane did not show the same pattern of results as objects rotated in depth. A one-tailed within-participant *t*-test on *d*-prime indicated that typically oriented objects (*M* = 2.86, *SD* = 0.98) were not better discriminated than atypically oriented objects (*M* = 2.77, *SD* = 0.96), *t*(19) = 0.71, *p* = 0.245, *dz* = 0.16; Figure 5). Moreover, participant performance was not more accurate for typically oriented objects (*M* = 0.86, *SD* = 0.11) than atypically oriented objects (*M* = 0.83, *SD* = 0.13), *t*(19) = 1.80 (two-tailed), *p* = 0.087, *dz* = 0.40. Interestingly though, response times were faster for typically oriented objects (*M* = 587 ms, *SD* = 110 ms) than atypically oriented objects (*M* = 599 ms, *SD* = 111 ms), *t*(19) = −25 (two-tailed), *p* = 0.037, *dz* = −0.50 (Figure 6), suggesting that the rotation in the picture plane did have some detrimental effect on performance. As in Experiment 1, typically oriented objects (*M* = 6.13, *SD* = 1.38) were rated as more highly typical viewpoints than atypically oriented objects (*M* = 2.37, *SD* = 1.18), significant in both parametric (*t*(19) = 7.19 (two-tailed), *p *< 0.001, *dz* = 1.61) and nonparametric (Wilcoxon signed rank test: *Z* = 4.13, *p *< 0.001) tests, again validating our classification of the viewpoints as more or less typical.

**Figure 7.**
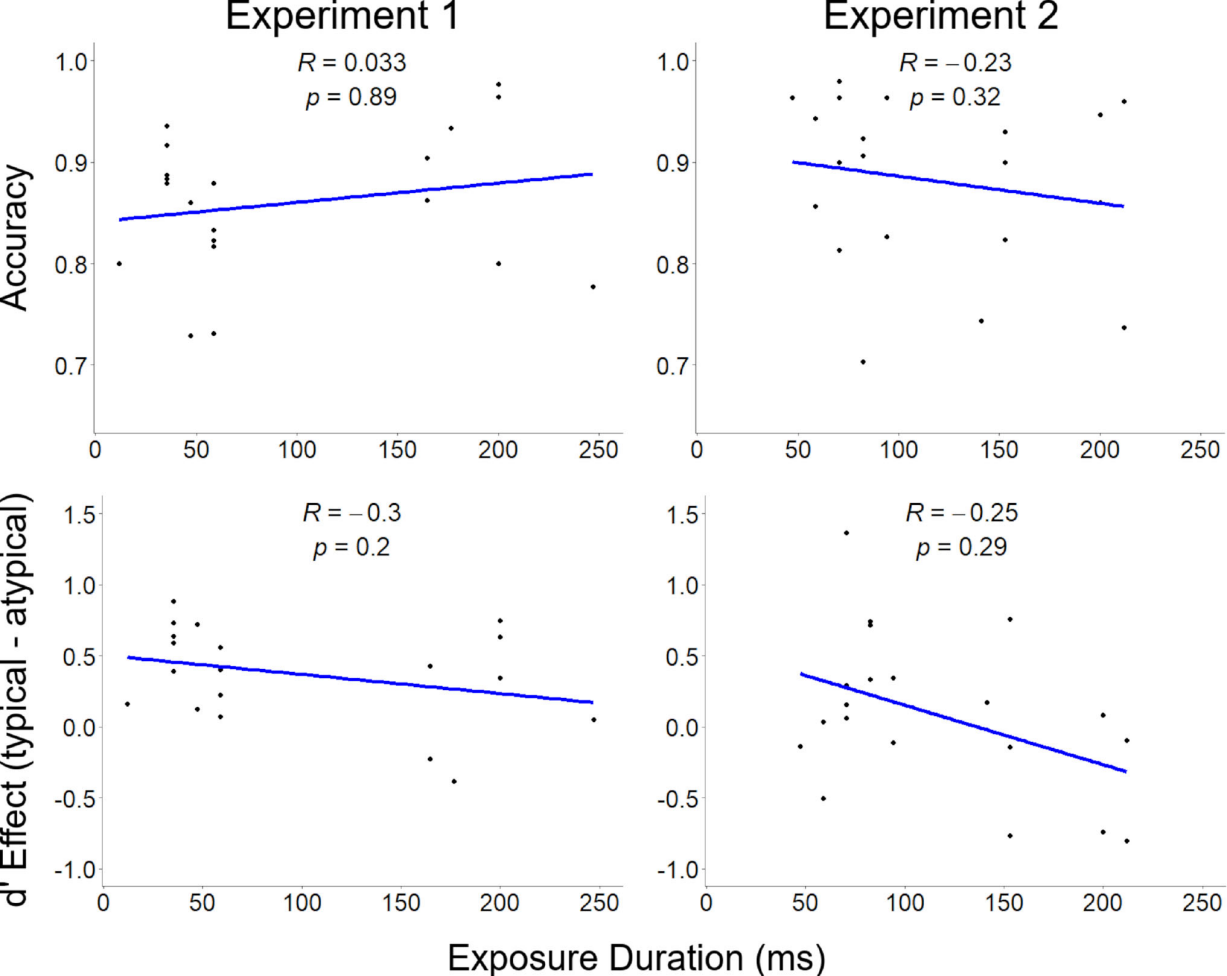
Relationship between stimulus exposure duration, accuracy (top row), and d-prime (bottom row), for Experiments 1 (left column) and 2 (right column). Spearman's rank correlation and associated *p* values are depicted at the top center of each facet.

Rating results from Experiment 2 indicate that participants assess typicality manipulation via rotations in the picture plane about as strongly as typicality manipulation via rotations in depth. Yet, results from the intact versus scrambled task indicate rotations in the picture plane did not produce comparable effects in discriminability to those of rotations in depth; the typicality effect from Experiment 1 (*dz* = 1.12, 95% confidence interval [CI] = 0.56 to 1.71), was of much greater magnitude than that of Experiment 2 (*dz* = 0.16, 95% CI = −0.29 to 0.61), although this did not reach the threshold for a statistical difference as the 95% CIs of the effect sizes overlapped. Quest-determined exposure durations were similar between Experiment 1 (*m* = 97 ms, *SD* = 76 ms) and Experiment 2 (*m* = 115 ms, *SD* = 57 ms), *t*(35.25) = 0.89, *p* = 0.38, *d* = 0.28, suggesting that differences in task difficulty between the experiments leading to differences in exposure durations were not responsible for the lack of a significant effect in Experiment 2. Figure 7 depicts the lack of a relationship between stimulus exposure duration, and accuracy and *d*-prime, respectively, for each experiment. Note the wide spreads of participants across y-axes regardless of exposure durations. Likewise, there were no significant differences between experiments in overall accuracy (Experiment 1: *m* = 0.86, *SD* = 0.07; Experiment 2: *m* = 0.88, *SD* = 0.08), *t*(36.94) = 0.92, *p* = 0.36, *d* = 0.29, hit rate (Experiment 1: *m* = 0.42, *SD* = 0.05; Experiment 2: *m* = 0.43, *SD* = 0.06), *t*(37.03) = 0.56, *p* = 0.58, *d* = 0.18), or false alarm rate (Experiment 1: *m* = 0.05, *SD* = 0.03; Experiment 2: *m* = 0.04, *SD* = 0.03), *t*(38) = 1.07, *p* = 0.29, *d* = 0.34). In short, Experiments 1 and 2 are comparable in all ways except that the typicality manipulation only impacted detection in Experiment 1 when the object was rotated in depth, and not when the object was rotated in the picture plane.

We note the possibility that there might exist a true effect of picture plane rotations that is smaller in magnitude than that of depth rotations and the current study is underpowered to detect such an effect. However, we are less interested in whether there might be a small effect of picture plane rotations, but whether this effect is smaller than that of a depth rotation. Such a result would suggest that our typicality effect may depend more on particular informative views, which are unchanged (with the exception of orientation) in the picture plane rotations. We thus replicate Experiment 2 with a larger sample size.

Recall that the 95% CIs for the effect sizes of Experiments 1 and 2 overlapped in our original samples. We wanted to power Experiment 3 to detect a difference between the effect of typicality in Experiment 1 and Experiment 3. In particular, we would need a 95% CI around the effect size of our follow-up experiment whose upper bound fell below the lower bound of that (*dz* = 0.56) of Experiment 1. Thus, here, we used power analysis as informed by the two one-sided *t*-test procedure (TOST) implemented in the “TOSTER” package in R statistical software (Lakens, 2018). Assuming a true Experiment 2 effect size of *dz* = 0, an alpha of 0.05, and 95% power, the power analysis indicated 43 participants would be required to show effect equivalence in the 0 to 0.55 interval, that is, below the lower bound of the confidence interval observed in Experiment 1. We note that this sample size does not achieve sufficient power to determine whether there is any effect of a picture plane rotation on detection. If the estimated effect size of Experiment 2 reflects the true effect, power analysis indicates that we would need to run 243 participants to achieve 80% power to detect a difference between typically oriented and picture plane rotation stimuli.

## Experiment 3: Replication and Bayesian analysis

We collected data from 46 participants, three of whom were excluded due to computer malfunctions. Again, a one-tailed within-participants *t*-test on *d*-prime indicated that typically oriented objects (*M* = 2.83, *SD* = 0.83) were not better discriminated than atypically oriented objects that were simply rotated in the picture-plane (*M* = 2.76, *SD* = 0.91), *t*(42) = 1.00, *p* = 0.161, *dz* = 0.15. Crucially, however, the CI observed here did not overlap with that of Experiment 1 (95% CI = -0.15 to 0.46]), falling outside the lower bound of Experiment 1’s interval at 0.56, which indicates that the typicality effect produced by picture plane rotations is truly smaller than that of depth rotations. Regarding whether we can provide support for a true effect of picture plane rotations at all, we must note that a null result under the frequentist approach to statistics cannot be taken as evidence for the null hypothesis. However, Bayesian hypothesis testing can directly evaluate evidence in favor of the null (e.g. see Wagenmakers, Verhagen, Ly, Matzke, Steingroever, Rouder, & Morey, 2017). We thus computed Bayes factors for our *d*-prime measures from each dataset, assigning Cauchy prior distributions to null hypotheses. We found very strong evidence (for background on the interpretation of Bayes factor magnitudes, see Jeffreys, 1961, and Lee & Wagenmakers, 2014) in favor of the alternative hypothesis (i.e. a difference between typical and atypical) in Experiment 1 (BF = 352.63) and moderate evidence in favor of the null in Experiment 2 (BF = 0.22). Furthermore, our 43 participants’ replication of Experiment 2 also found moderate evidence in favor of the null hypothesis (BF = 0.19), consistent with a combined measure using the full 63 participants from both the original and replication studies (BF = 0.21). In short, picture-plane rotations not only produced a smaller typicality effect than depth rotations, but the evidence favored no difference between the detection of typically orientated objects and objects rotated in the picture plane. A similar lack of an effect of inversion on detection sensitivity was observed by Mack, Gauthier, Sadr, and Palmeri (2008).

Comparing discriminability effects between depth rotations (Experiment 1) and picture-plane rotations (Experiments 2 and 3) suggests that the frequency with which we typically encounter an object viewpoint is perhaps not the driving factor in producing perceptual difficulties and instead suggests a more interesting possibility: that our results may be better described as reflecting canonical and non-canonical viewpoints, a factor that depends more on informativeness (i.e. the presence of salient information about the identity of the object; Palmer, Rosch & Chase, 1981) than frequency of occurrence.

## General discussion

In these experiments, we predicted that typical, and thus more recognizable, object viewpoints would be more easily discriminated from noise than their less recognizable counterparts, and this is despite the fact that our task does not require identification of the objects. Our prediction was correct in the case of depth rotations. Atypical viewpoints of an object were less readily discriminated from scrambled images than typical ones. These data are in keeping with Palmer, Rosch, and Chase's (1981) original proposal that a canonical perspective of an object should be more readily perceived. Unlike earlier work, however, here, our task did not require identification, placing this result in the perceptual domain rather than in a later cognitive processing stage (Pylyshyn, 1999).

Much of the significance of the present findings derive from the nature of our task, and although its phenomenology is convincing, critics without direct experience of the task might feel tempted to argue that it is no different than those commonly used in the object perception literature. Crucially, rather than relying on explicit identification or categorization of objects as previous work has done, we only required participants to respond whether their perception of a briefly presented stimulus was more akin to an object or noise. In this sense, discriminating objects from noise is better described as a detection task, rather than a conventional discrimination task wherein stimuli are to be binned into extant, socially agreed-upon categories. Here, it is merely the case that on some trials the target stimulus is present, whereas on others it is not. Set to proper presentation duration thresholds, participants may then detect targets (hit), fail to detect targets (miss), respond that no target was present when it was truly absent (correct rejection), or respond that a target was present even though it was truly absent (false alarm), all of which occurred with varying frequency within our experiments. Intriguingly, aspects related to the identity of the target influenced its subsequent detection, even though participants were never asked to explicitly identify the target, and from anecdotal descriptions, often lacked any coherent guess as to what that target's identity could have been.

### Confound analysis

Because our typical and atypical stimuli potentially differed in many dimensions, and their corresponding diffeomorphs and masks could have inherited some of those differences, it is important to confirm that the effects that we observe truly stem from an object’s canonicity rather than more trivial causes. If differences in *d*-prime between typical and atypical stimuli were driven by false alarms rather than hits, for instance, we could have reason for concern that atypical diffeomorphs systematically differed from their typical counterparts, thereby causing participants to more frequently false alarm to one class. Upon analysis, we find that differences in false alarm rates between typical and atypical conditions did not differ in either Experiment 1 (typical: *m* = 0.09, *SD* = 0.06; atypical: *m* = 0.10, *SD* = 0.07; *t*(19) = -0.70, *p* = 0.49, *d* = 0.16), Experiment 2 (typical: *m* = 0.08, *SD* = 0.06; atypical: *m* = 0.07, *SD* = 0.07; *t*(19) = 0.64, *p* = 0.53, *d* = 0.14), or Experiment 3 (typical: *m* = 0.08, *SD* = 0.07; atypical: *m* = 0.07, *SD* = 0.07; *t*(42) = 0.59, *p* = 0.56, *d* = 0.09).

Another potential confound comes from the possibility of differences in the total amount of image-defining pixels between typical object viewpoints and their atypical counterparts. If canonical viewpoints contained more image-defining pixels than non-canonical viewpoints, then perhaps the difference in hit rate derives not from canonicity, but instead raw stimulus energy. Note that this concern is only relevant for Experiment 1 because the picture plane rotation manipulation performed in Experiment 2 preserved the same number of pixels between stimulus classes. We can quantify the number of image-defining pixels by counting the number of white (i.e. background) pixels present in each image from Experiment 1. To measure the contribution of this pixel factor to hit rate, we constructed a multilevel logistic model to estimate the likelihood of a hit using an object's viewpoint (typical or atypical), the number of background pixels in the stimulus, and their interaction, as predictors terms, respectively, with a random intercept terms for both participant and object identity. The model revealed a modest effect of the number of background pixels on detection (odds ratio = 1.17, 95% CI = 1.07 to 1.29), but with a much stronger effect of object viewpoint that explained variance over and above that accounted for by background pixels (odds ratio = 1.71, 95% CI = 1.49 to 1.95), and, critically, there was no interaction between the two predictors (odds ratio = 1.01, 95% CI = 0.88 to 1.16). Thus, although in Experiment 1, typical objects (*m* = 154,690, *SD* = 27,687) did contain fewer background pixels on average than its atypical objects (*m* = 160,287, *SD* = 30,597), the effect size was rather small, *t*(179) = 3.18, *p* = 0.002, *d* = 0.19 and did not drive our typicality effect. Indeed, the pixel differences were in the opposite direction from what one might predict would influence detection; that is, the less detectable stimulus had more stimulus energy. Overall, we find strong evidence of an effect of typicality of viewpoint on object detection in Experiment 1 and null or negligible effects deriving from other factors.

### Deconstructing “recognizability”

We predicted that the picture plane rotations, by virtue of being encountered infrequently in the real world, would be less likely to make contact with existing representations of real-world statistical regularities, originally assuming that the brain would over time construct statistically regular representations on the basis of how frequently certain stimuli are encountered in daily life. The observed pattern of data, however, suggests the situation may be more nuanced. We did not observe a significant difference in discriminability between typical views and atypical rotations in the picture plane (although we did observe faster reaction times for picture plane rotated objects) in either Experiments 2 or 3. Post hoc Bayesian analysis offered additional support for the lack of a true difference in discriminability for picture plane rotated objects. Furthermore, Experiment 3, which was better powered than Experiment 2, showed that picture-plane rotations produced a significantly smaller effect of typicality than depth rotations. Such a result is consistent with role for canonicity in detection.

Although our typical views are encountered more frequently, they also tend to be more canonical; that is, they also display the critical information needed to quickly recognize the object. To be clear, however, we make no claim that categorization precedes or co-occurs with discrimination in our task. We only posit that more informative views allow our participants to better recover coherence from noise. In fact, it is likely that the canonical orientations are more typical (or more frequently predicted) precisely because they are more informative. For instance, objects are often photographed from canonical perspectives. Objects rotated in depth, on the other hand, tend to have more shadows and fewer object defining pixels than their typical counterparts. This is not true of picture plane rotations as objects rotated in the picture plane still preserve all the critical information needed to resolve their identity. Thus, despite being encountered infrequently in the real world, one could argue that picture plane rotations are still more canonical than depth rotations. In other words, it is not frequency, per se, that is driving the perceptual advantage for typical viewpoints, but canonicity.

Our findings in terms of picture plane rotations might seem to contrast with previous findings at first glance. Jolicoeur and colleagues found consistent detriments in identification speed and accuracy as images were rotated further from their starting axis (Jolicoeur, 1985; Lawson & Jolicoeur, 1998; Lawson & Jolicoeur, 1999; Lawson & Jolicoeur, 2003). If, however, identification draws from higher order cognitive processes, as Pylyshyn (1999) argues, then our diverging findings point to effects that take place at different levels of the visual processing hierarchy. In other words, given a cognitive task, rotating images along the picture plane produces significant disruptions, however, when the task is perceptual in nature, the impact is much more subtle. Peterson and Gibson (1994), on the other hand, do use a task that is arguably more perceptual in nature than cognitive via their figure-ground segregation task, and nonetheless they report effects of picture-plane rotations. We reason that key differences between our stimulus sets can explain this discrepancy, however; namely, that Peterson and Gibson used two-toned stimuli whose only defining feature is a border separating two regions, whereas our stimuli, whereas not at the level of photographic realism, are still realistic and feature-rich. This difference could feasibly produce the result that rotating an impoverished two-toned stimulus by 180 degrees strips it of all meaning whereas performing the same rotation on a feature-rich stimulus (particularly given that the same information from the typical view is easily recoverable) preserves its meaning and allows for competitive performance with its upright counterpart.

Taking the current results together with the literature (Greene, Botros, Beck, & Fei-Fei, 2015; Caddigan et al., 2017; Lupyan, 2017; Smith & Loschky, 2019), it is perhaps more useful to conceive of recognizability as reflecting our ability to predict identity on the basis of past experience. We hypothesize that the greater sensitivity to “recognizable” images depends upon gradually constructed, continuously updated, oftentimes implicit representations of real-world statistical regularities; that is, over the course of our lifetime, our brains learn which visual features, objects, scenes, and events are not only likely to occur, but also render the visual world more meaningful. Thus, when we speak of real-world statistical regularities here, we do not imply the types of statistical regularities described in the statistical learning literature which develop over the course of an experiment, but instead, over the lifetime. We suggest that our results, along with earlier results, demonstrate that the brain is not only sensitive to real-world statistical regularities but makes good use of them, allowing us to more readily perceive items that conform to the patterns engrained in our neural architecture.

### Ties to predictive coding

The concept of a real-world statistical regularity is critical to predictive coding frameworks (Rao & Ballard, 1999; Friston, 2005; Friston, 2008; Spratling, 2008) in which such representations serve as predictions with which to compare against the current input, allowing the brain to make sense of the busy visual world more efficiently. These theories describe not only the evolution of short-term network dynamics such as the changing of firing rates as an individual stimulus or a stimulus series is processed, but also long-term network dynamics such as the tuning of synapse weights throughout network hierarchies as models of the world are updated, and it is the latter of these processes we invoke in interpreting the present results.

In keeping with this framework, this study is one of a growing number of studies that have found that more predictable stimuli, or globally statistically regular stimuli (as opposed to those established within the context of the experiment), are more readily perceived than less predictable or statistically irregular ones (Greene et al., 2015; Caddigan et al., 2017; Lupyan, 2017; Smith & Loschky, 2019). For instance, Greene et al. (2015) used the same intact/scrambled design with stimuli comprised of either probable or improbable events. Improbable events were again less discriminable from noise than their probable counterparts, demonstrating that stimulus probability (defined not within an experiment but over an observer's lifetime), independent of category, influences perceptibility. Within this same theme, Smith and Loschky (2019) found that target images in predictable sequences (based on movements through a familiar space) were better discriminated from noise than those in random sequences. More recently, we extended the intact/scrambled paradigm used in the present set of experiments to familiar and novel logos or faces (Yang & Beck, in preparation). Both famous logos and faces were discriminated from noise more readily than novel ones. This result is particularly challenging to detection-first models (Pylyshyn, 1999) since all faces are easily recognized as faces, yet participants do so with shorter durations for familiar than novel faces.

Across these experiments, using a variety of mask types and stimulus classes, participants were quicker to perceive the presence of a statistically regular image, that is, the more predictable image, based on long-term real-world experience. The candidate model structure most consistent with these findings is a recursive model where processing in a hierarchically superior brain area is capable of biasing ongoing processing in a hierarchically inferior one. Popular examples of this type of model are the aforementioned hierarchical predictive coding models (Rao & Ballard, 1999; Friston, 2005; Friston, 2008; Spratling, 2008), which often characterize the brain as an inference machine, constantly drawing predictions about incoming signals and iteratively updating predictions across multiple levels of the processing hierarchy as new information comes to light. Embedded within these models is an assumption that each brain region is capable of representing learned real-world statistical regularities abstracted from the environment. Representations of real-world statistical regularities then perform most of the “work” of perception by predicting incoming signals, whereas feedforward processing is tasked primarily with propagating forward the residual errors of predictions, allowing for recursive updating of predictions until residual errors flatten toward zero.

Our results would suggest that perceptual predictions are shaped, in part, by a conceptual framework that is based on more than mere exposure, and that the success of such predictions in detecting the presence of an object hinges on the availability of canonical information tightly linked to object identity. In other words, we should think of real-world statistical regularities as statistical priors in the Bayesian sense; the brain makes predictions based on past experiences designed to minimize prediction error with respect to recognition. Consequently, predictive processes would succeed based on the presence of specific information, and the magnitude of effects produced by real-world statistical regularity manipulations could be predicted by the amount of new information present in an exemplar that does not conform to the brain's canonical stored representation.

Whereas Experiments 2 and 3 cannot rule out a role for frequency in establishing statistical regularities more generally, they do indicate that picture plane rotations produce less robust decrements in perception than depth rotations, suggesting that representations of real-world statistical regularity with respect to object orientations are more strongly shaped by how informative a particular view is to the identity of the object than by the frequency with which the view is encountered. Although objects rotated in the picture plane preserved all the critical features needed to resolve their identity, they still might require some mental rotation to be matched to the canonical viewpoint. Thus, the savings in *d*-prime and the drop in response time could indicate participants are grasping key features, recovering object identity, and then mentally rotating objects to fit their templates before giving a response, consistent with Palmer et al.’s (1981) findings on object naming times for rotated objects and Blanz, Bülthoff, and Tarr's (1999) findings on object canonicity.

## Conclusions

We have demonstrated that objects rotated in depth show similar drops in discriminability to those of bad exemplars of natural scenes. Reducing the recognizability of an object by changing its viewpoint obscures not only our ability to identify it, but more fundamentally, our ability to detect that an intact object is there at all, suggesting that an object's identity can affect basic perceptual processes. These experiments add to an increasing literature showing that statistically regular images are more readily perceived than irregular ones.
